# Role of Dexamethasone in Reducing Postoperative Pain Following Cardiac Surgery: A Single-Center Retrospective Cohort Study

**DOI:** 10.7759/cureus.88707

**Published:** 2025-07-24

**Authors:** Said K, R Seddiki, I Serghini

**Affiliations:** 1 Emergency Medicine, Mohamed V Training Military Hospital, Rabat, MAR; 2 Anesthesiology, Hassan II Military Hospital, Marrakech, MAR; 3 Anesthesiology, Avicenna Military Hospital, Marrakech, MAR

**Keywords:** cardiac surgery, chronic pain, dexamethasone, opioid-sparing, postoperative pain, retrospective study, visual analog scale

## Abstract

Background: Optimal management of postoperative pain after cardiac surgery remains a clinical priority. Glucocorticoids such as dexamethasone may enhance analgesia and reduce opioid requirements, but evidence specific to cardiac surgery is limited and mixed.

Objective: This study aimed to evaluate the effect of a single intraoperative dose of dexamethasone on early postoperative opioid consumption, pain intensity, and chronic postoperative pain incidence following elective cardiac surgery.

Methods: This retrospective observational study was conducted at Avicenna Military Hospital (Marrakech, MAR), a single tertiary center, from January 1, 202,2 to December 31, 2023. A total of 45 adult patients undergoing elective cardiac surgery via median sternotomy were included. Of whom, 22 received IV dexamethasone (0.1-0.2 mg/kg) after induction, while 23 received no corticosteroids. All patients received standardized multimodal analgesia with IV paracetamol and morphine via patient-controlled analgesia (PCA). Primary outcomes were cumulative IV morphine use on postoperative day one (POD1) and POD3. Secondary outcomes included pain scores (visual analog scale (VAS) 0-10) on POD1 and POD3 and chronic post-sternotomy pain at three months.

Results: Dexamethasone significantly reduced morphine consumption on POD1 (12.2 ± 2.9 mg vs. 18.5 ± 3.8 mg, p < 0.001) and POD3 (6.1 ± 2.1 mg vs. 9.2 ± 2.7 mg, p < 0.001). The VAS scores were also lower in the dexamethasone group on POD1 (3.5 ± 1.0 vs. 5.6 ± 1.1, p < 0.001) and POD3 (2.0 ± 0.8 vs. 3.5 ± 1.0, p < 0.001). Chronic pain was reported in 18.2% of the dexamethasone group vs. 39.1% in controls at three months (risk difference -20.9%, p = 0.09), a clinically relevant trend despite not reaching statistical significance.

Conclusion: A single intraoperative dose of dexamethasone significantly reduces early postoperative opioid use and pain intensity in cardiac surgery. Although the reduction in chronic pain did not reach statistical significance, the observed trend suggests a potential long-term benefit. Further randomized trials with larger sample sizes are warranted to confirm these findings.

## Introduction

Postoperative pain following cardiac surgery via median sternotomy remains a major clinical concern. Inadequate pain control is associated with impaired respiratory function, delayed ambulation, prolonged ICU stay, and a higher risk of developing chronic post-sternotomy pain syndromes [1,2]. Current pain management strategies include opioids, regional anesthesia, and adjuncts such as ketamine or gabapentinoids, yet their limitations-such as respiratory depression, hemodynamic instability, or delirium-necessitate exploring alternative agents [3,4].

Glucocorticoids, particularly dexamethasone, exhibit potent anti-inflammatory and analgesic effects. In non-cardiac surgery, dexamethasone has been shown to reduce postoperative pain, nausea, and opioid consumption without increasing complications [5,6]. In cardiac surgery, its role has been less extensively studied, with most trials-such as the Dexamethasone for Cardiac Surgery (DECS) trial-focusing primarily on inflammatory biomarkers and major morbidity rather than analgesic endpoints [7].

This retrospective cohort study investigates whether a single intraoperative dose of dexamethasone can reduce acute postoperative pain, measured by morphine consumption and VAS scores on postoperative days 1 and 3, and decrease the incidence of chronic pain at 3 months. The goal is to clarify dexamethasone’s potential role in optimizing multimodal analgesia protocols in cardiac surgery.

## Materials and methods

Study design and patients

This retrospective observational study was conducted at Avicenna Military Hospital, a tertiary referral center for adult cardiothoracic surgery in Marrakech, Morocco. The study included adult patients who underwent elective cardiac surgery via median sternotomy between January 1, 2022, and December 31, 2023. Eligible procedures encompassed isolated or combined coronary artery bypass grafting (CABG), performed either on-pump or off-pump; valve surgeries (aortic, mitral, or tricuspid replacement or repair); and ascending aortic interventions. All surgeries were performed by a senior surgical team, following standardized institutional protocols for anesthesia and analgesia management.

Patients in the intervention group received a single IV dose of dexamethasone (0.1-0.2 mg/kg) after induction of general anesthesia and before initiation of cardiopulmonary bypass. The control group received no perioperative corticosteroids. Intraoperative and postoperative data were collected, including detailed anesthetic records, postoperative morphine consumption on days one and three, and pain scores assessed using the VAS.

A total of 57 patients were screened, and the final cohort comprised 45 patients, divided into a dexamethasone group (n = 22) and a control group (n = 23), based on intraoperative corticosteroid exposure. Institutional Review Board approval was obtained prior to study initiation. Given the retrospective design, the requirement for individual informed consent was waived. All study procedures complied with the ethical principles outlined in the Declaration of Helsinki.

Inclusion criteria

This study included adult patients aged 18 years and older who were scheduled for elective cardiac surgery through a median sternotomy. Eligible surgical procedures encompassed isolated or combined on-pump or off-pump CABG, as well as valve surgeries involving the aortic, mitral, or tricuspid valves, including both repair and replacement. Combined procedures, such as CABG with concomitant valve surgery, were also included. Only patients with complete intraoperative anesthesia records and comprehensive postoperative analgesia data, specifically morphine consumption and pain scores, were considered for analysis.

Exclusion criteria

Patients were excluded if they underwent redo-sternotomy procedures or were chronic opioid users, defined as those receiving more than 30 mg of morphine equivalents daily for over 30 days prior to surgery. Additional exclusion criteria included long-term corticosteroid or immunosuppressive therapy, known allergy or contraindication to dexamethasone, intraoperative death, or the need for surgical re-exploration within 24 hours postoperatively. Patients with incomplete medical records that prevented evaluation of the primary or secondary outcomes were also excluded.

Final cohort and group allocation

Of the 57 patients screened, 12 were excluded: seven for chronic opioid use, three for ongoing corticosteroid or immunosuppressive therapy, and two for incomplete postoperative records. The final cohort comprised 45 patients who met all inclusion criteria and were eligible for analysis. Based on a retrospective review of intraoperative anesthesia records, patients were categorized into two groups according to the dexamethasone dose received during surgery.

Intraoperative intervention and group definitions

The dexamethasone group (n = 22) received a single IV dose of dexamethasone (0.1-0.2 mg/kg), administered immediately after induction of general anesthesia and before skin incision. The dose was weight-adjusted and selected by the attending anesthesiologist according to institutional protocols. In contrast, the control group (n = 23) received standard intraoperative care without any exposure to perioperative corticosteroids.

Standardized analgesic and perioperative management

**Figure 1:**
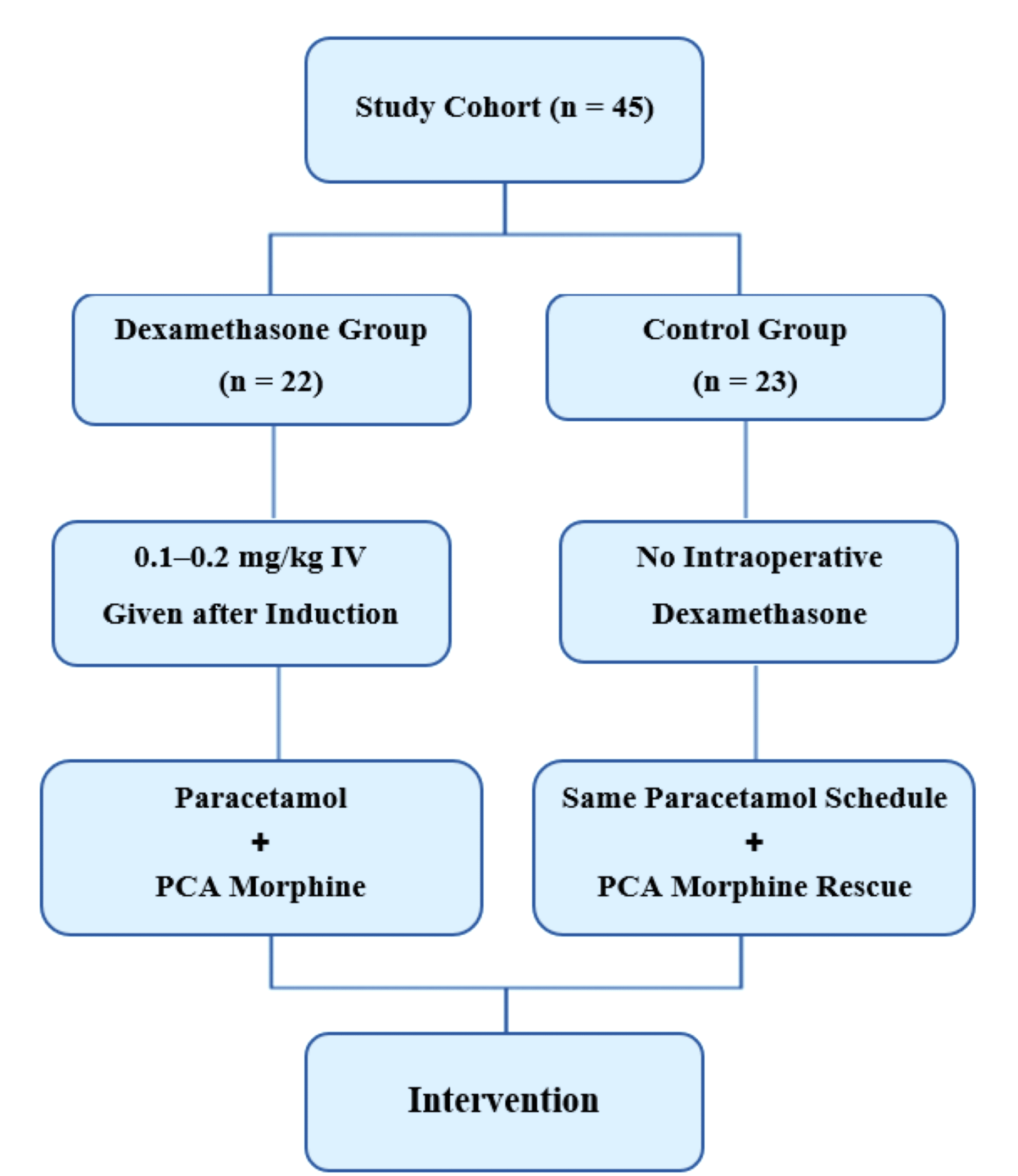
Study flowchart showing allocation to dexamethasone (n = 22) and control (n = 23) groups, with both receiving standardized postoperative paracetamol and PCA morphine. PCA: Patient-controlled analgesia

All patients received the same standardized multimodal analgesic regimen: IV paracetamol 1 gm every six hours starting at the end of surgery and continued for a minimum of 72 hours postoperatively. Patient-controlled analgesia (PCA) with morphine was used for breakthrough pain, programmed uniformly (e.g., 1 mg bolus, five- to 10-minute lockout, no background infusion). Non-steroidal anti-inflammatory drugs (NSAIDs) were avoided due to institutional contraindications, particularly in cardiopulmonary bypass (CPB) cases. Anesthetic technique, intraoperative monitoring, and hemodynamic targets were applied consistently across all patients. Postoperative care was delivered in a common ICU setting, ensuring uniformity in pain assessment and analgesia administration (Figure 1).

Definition of outcomes

The primary and secondary endpoints were defined a priori and assessed using standardized measures. The primary outcome was cumulative IV morphine-equivalent consumption at 24 hours postoperatively (postoperative day one (POD1)) and 72 hours (POD3). Secondary outcomes included patient-reported pain intensity, measured by a 10-point VAS on POD1 and POD3, and the presence of chronic post-sternotomy pain at three months. Chronic pain was defined as new or persistent thoracic discomfort requiring analgesic treatment or interfering with daily activity and was assessed during routine outpatient follow-up or via structured telephone interview (Table 1).

**Table 1:** Primary outcome and secondary outcomes POD: Postoperative day, VAS: Visual analog scale

Outcome	Definition and assessment time-point
Primary	24-hour cumulative IV morphine-equivalent dose on POD1 and POD3
Secondary	• Pain intensity on a 0-10 VAS on POD1 and POD3. • Presence of chronic post-sternotomy pain at three months, assessed in follow-up clinic or by structured phone interview. 'Chronic pain' is defined as persistent or new-onset thoracic pain requiring medication or impairing daily function.

Data collection

Comprehensive data were systematically collected for all enrolled patients using a standardized electronic data-extraction form developed a priori and pilot-tested prior to study initiation. Two independent investigators (blinded to group allocation) performed data extraction in parallel to minimize selection bias and ensure accuracy. Any discrepancies between reviewers were adjudicated through joint discussion and resolved by consensus; when necessary, a third senior reviewer was consulted for arbitration.

Demographic data included age (years), sex, BMI, and relevant comorbidities (e.g., diabetes mellitus, hypertension, coronary artery disease), all of which were recorded from the institutional electronic medical record system. Operative variables comprised the type of cardiac surgical procedure performed (e.g., isolated CABG, valve surgery, or combined procedures), total duration of surgery (minutes), CPB time (minutes), and aortic cross-clamp time (minutes). These data were extracted directly from anesthetic charts and operative reports validated by the attending surgical and anesthesiology teams.

Postoperative analgesic consumption was rigorously documented, with morphine administration recorded in milligrams and verified through medication administration records within the first 72 hours postoperatively. For standardization, all opioid doses were converted to IV morphine equivalents where applicable. Pain intensity was assessed using the VAS (range 0-10) recorded at regular intervals by trained nursing staff as part of routine clinical care and extracted from the postoperative monitoring chart at two prespecified time points: POD1 and POD3.

Chronic pain status at the three-month follow-up was assessed via structured telephone interviews conducted by investigators blinded to group allocation, using a predefined questionnaire. Chronic postoperative pain was defined as pain at or near the surgical site persisting for more than three months after the index procedure, not attributable to other causes (e.g., infection, malignancy). All responses were cross-validated with outpatient follow-up notes when available.

All data were entered into a secure, password-protected database with audit trails. Data integrity was verified via random sampling and double-entry checks to ensure accuracy prior to statistical analysis.

Statistical analysis

All statistical analyses were performed using Python version 3.10 (Python Software Foundation, Wilmington, DE, USA) with relevant packages, including pandas, numpy, and scipy.stats. Data were analyzed on an intention-to-treat basis. Prior to statistical testing, all continuous variables were examined for distributional properties using the Shapiro-Wilk test for normality and visual inspection of histograms and Q-Q plots. Homogeneity of variance was assessed using Levene’s test when applicable.

Continuous variables are summarized as mean ± standard deviation (SD) when normally distributed, or as median and interquartile range (IQR) for non-normal distributions. Between-group comparisons of normally distributed continuous variables (e.g., morphine consumption, VAS pain scores) were performed using the unpaired two-sample (independent) Student’s t-test. For non-normally distributed variables, the Mann-Whitney U test was used instead, though this was not required for the main outcomes as normality criteria were met.

Categorical variables (e.g., sex, incidence of chronic postoperative pain) are presented as frequencies and percentages. Group comparisons for categorical variables were conducted using the chi-square (χ²) test when cell counts permitted. When the expected frequency in any contingency table cell was <5, Fisher’s exact test was used to ensure statistical validity.

Effect sizes were calculated for all primary outcomes. For continuous outcomes, the absolute between-group differences were reported with corresponding 95% confidence intervals (CIs). For categorical outcomes, absolute risk differences and relative risk reductions were calculated, also with 95% CIs. Where appropriate, relative percent reductions were additionally reported to contextualize clinical significance (e.g., percentage reduction in opioid use).

All statistical tests were two-sided, and a p-value < 0.05 was considered statistically significant. No correction was applied for multiple comparisons, given the prespecified and limited number of primary and secondary endpoints. Missing data were minimal and did not exceed 5% for any variable. No imputation was performed as data were assumed to be missing at random, and the analytic approach was complete case analysis. Data visualization, including bar plots, line graphs, and error bars, was performed using the matplotlib and seaborn libraries. Figures were designed to enhance the clarity of between-group trends and include 95% CI or standard deviation indicators where relevant.

## Results

Baseline characteristics

A total of 45 patients undergoing elective cardiac surgery were included (22 in the dexamethasone group and 23 in the control group). The two groups were comparable across all key demographic and intraoperative variables. There were no statistically significant differences in mean age (64.3 ± 7.8 vs. 65.1 ± 8.2 years, t = -0.34, p = 0.73), sex distribution (male: 81.8% vs. 82.6%, χ² = 0.01, p = 0.93), or BMI (27.1 ± 3.5 vs. 26.9 ± 3.7 kg/m², t = 0.19, p = 0.85). Surgical characteristics were also similar between groups, including procedure type (CABG only: 59.1% vs. 60.9%, χ² = 0.01, p = 0.91), CPB duration (92 ± 21 vs. 95 ± 24 minutes, t = -0.47, p = 0.64), and aortic cross-clamp time (68 ± 15 vs. 70 ± 16 minutes, t = -0.44, p = 0.66). The full details are presented in Table 2.

**Table 2:** Baseline characteristics of study groups Continuous variables were compared using independent samples t-tests, and categorical variables using the chi-square (χ²) test. Significance was set at p < 0.05. CABG: Coronary artery bypass grafting

Variable	Dexamethasone (n = 22)	Control (n = 23)	Test statistic	p-value
Age, years (mean ± SD)	64.3 ± 7.8	65.1 ± 8.2	t = -0.34	0.73
Sex, male n (%)	18 (81.8%)	19 (82.6%)	χ² = 0.01	0.93
BMI, kg/m² (mean ± SD)	27.1 ± 3.5	26.9 ± 3.7	t = 0.19	0.85
Procedure type: CABG only	13 (59.1%)	14 (60.9%)	χ² = 0.01	0.91
Procedure type: Valve ± CABG	9 (40.9%)	9 (39.1%)	χ² = 0.01	0.91
CPB duration, min (mean ± SD)	92 ± 21	95 ± 24	t = -0.47	0.64
Aortic cross-clamp time, min (mean ± SD)	68 ± 15	70 ± 16	t = -0.44	0.66

Primary outcome

Early Postoperative Opioid Consumption

Intraoperative administration of dexamethasone was associated with a marked and statistically significant reduction in postoperative opioid requirements. On POD1, patients in the dexamethasone group required 12.2 mg of morphine (SD 2.9), compared to 18.5 mg (SD 3.8) in the control group, an absolute difference of -6.3 mg (95% CI, -8.3 to -4.3; t = -6.16, p < 0.001), corresponding to a 34% relative reduction. This opioid-sparing effect persisted through POD3, with morphine consumption averaging 6.1 mg (SD 2.1) in the dexamethasone group versus 9.2 mg (SD 2.7) in controls (difference -3.1 mg; 95% CI, -4.5 to -1.7; t = -4.34, p < 0.001). These findings are summarized in Table 3.

**Table 3:** Postoperative opioid consumption and pain scores Continuous variables were compared using independent samples t-tests. A p-value < 0.05 was considered statistically significant. POD: Postoperative day, VAS: Visual analog scale

Variable	Dexamethasone (n = 22)	Control (n = 23)	Test statistic	p-value
Morphine POD1, mg	12.2 ± 2.9	18.5 ± 3.8	t = –6.16	< 0.001
Morphine POD3, mg	6.1 ± 2.1	9.2 ± 2.7	t = –4.34	< 0.001
VAS POD1	3.5 ± 1.0	5.6 ± 1.1	t = –6.44	< 0.001
VAS POD3	2.0 ± 0.8	3.5 ± 1.0	t = –5.48	< 0.001

Secondary outcome

Early Postoperative Pain Intensity

**Figure 2:**
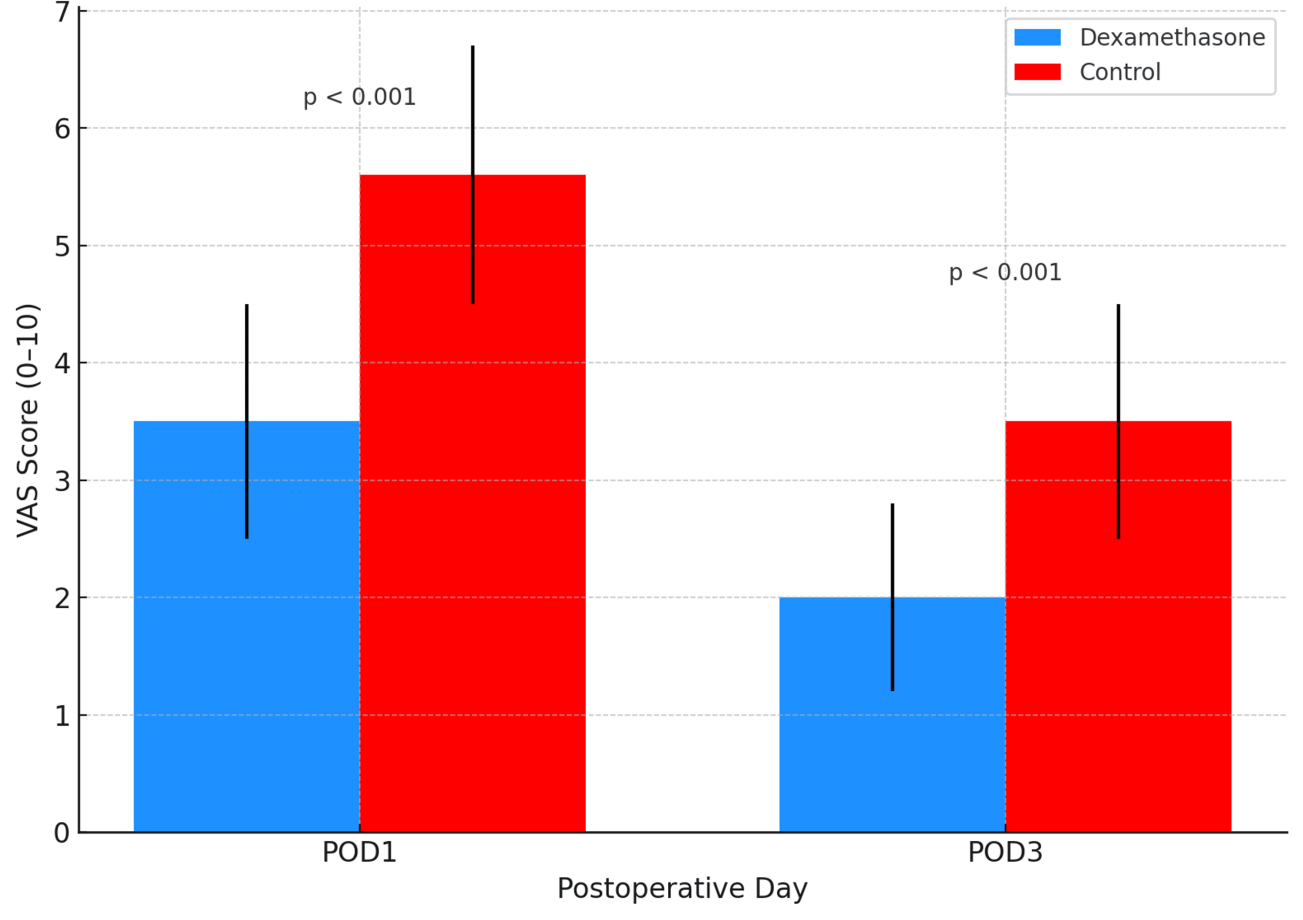
VAS pain scores on POD1 and POD3 by group (p < 0.001) VAS: Visual analog scale, POD: Postoperative day

Reductions in opioid consumption were mirrored by significantly lower pain intensity scores in the dexamethasone group. On POD1, the mean VAS score was 3.5 (SD 1.0) compared with 5.6 (SD 1.1) in the control group, an absolute difference of -2.1 points (95% CI, -2.7 to -1.5; t = -6.44, p < 0.001). On POD3, mean VAS scores remained significantly lower at 2.0 (SD 0.8) versus 3.5 (SD 1.0), yielding a difference of -1.5 points (95% CI, -2.0 to -1.0; t = -5.48, p < 0.001). These results are illustrated in Figure 2. Together, these findings confirm that a single intraoperative dose of dexamethasone significantly reduced both opioid consumption and subjective pain intensity during the early postoperative period.

Chronic Post-sternotomy Pain at the Three-Month Follow-Up

At the three-month follow-up, chronic postoperative pain was reported in four out of 22 patients (18.2%) in the dexamethasone group, compared with nine out of 23 patients (39.1%) in the control group. This corresponds to an absolute risk reduction of 20.9% and a relative risk reduction of approximately 53%, favoring dexamethasone. Although this difference did not reach conventional statistical significance (χ² = 2.84, p = 0.09), the trend toward reduced chronic pain was consistent and clinically notable. The 95% confidence interval for the absolute risk difference (-45.5% to +3.6%) suggests that, while uncertainty remains due to sample size limitations, the true effect could be substantial. The lower boundary of the CI indicates a meaningful benefit, and the upper boundary barely crosses the null, highlighting a possible type II error due to limited power. From a clinical perspective, reducing persistent post-sternotomy pain, a condition known to affect quality of life and rehabilitation, by even a modest margin is highly valuable. These findings justify further investigation in larger randomized trials powered specifically to evaluate long-term analgesic outcomes.

## Discussion

In this retrospective single-center cohort, intraoperative administration of a single dose of dexamethasone (0.1-0.2 mg/kg) immediately following anesthesia induction was associated with a marked improvement in postoperative analgesic outcomes among patients undergoing elective cardiac surgery (see Appendix A). Specifically, dexamethasone recipients experienced a statistically significant reduction in cumulative morphine use on both POD1 and POD3, with a relative reduction of approximately 34%. This opioid-sparing effect was paralleled by significantly lower pain intensity, as measured by the VAS, at both time points. These findings indicate that dexamethasone conferred a consistent and clinically relevant analgesic benefit in the early postoperative period.

Although the difference in chronic post-sternotomy pain at three months did not achieve statistical significance (18.2% vs. 39.1%, p = 0.09; χ² = 2.84), the absolute risk reduction of -20.9% and a relative reduction exceeding 50% are suggestive of a potentially meaningful long-term benefit. The 95% CI (-45.5% to +3.6%) narrowly crosses the null, likely reflecting a type II error due to limited sample size rather than a true absence of effect. From a clinical standpoint, even a modest reduction in persistent post-sternotomy pain, known to impair rehabilitation and long-term quality of life, can be highly impactful.

These results are in line with a growing body of literature supporting the perioperative use of corticosteroids for analgesia in major surgical procedures. Large observational studies in cardiac surgery populations have shown that dexamethasone can reduce early opioid consumption by nearly 30%, though without significant effects on broader functional outcomes such as post-discharge recovery time [8]. Systematic reviews and meta-analyses across diverse surgical settings similarly demonstrate that glucocorticoids are associated with decreased opioid use and pain intensity, without increasing the risk of surgical site infections or wound complications [9].

Importantly, while landmark trials such as the DECS study confirmed the anti-inflammatory and organ-protective effects of dexamethasone in cardiac surgery, they did not assess its impact on postoperative pain or opioid utilization [7]. Studies that failed to demonstrate analgesic efficacy frequently administered dexamethasone too late in the perioperative course, often after CPB initiation, thereby potentially missing the preemptive anti-inflammatory window recommended by contemporary multimodal analgesia guidelines [10,11]. Our protocol, which involved administering dexamethasone prior to skin incision, aligns with this pharmacologic timing principle and may explain the observed analgesic efficacy.

Moreover, our VAS score reductions align closely with those reported in a recent meta-analysis, where systemic dexamethasone was shown to lower early postoperative pain with a standardized mean difference of -0.6 at 24 hours and -0.4 at 48 hours across multiple surgical fields [6]. Beyond early pain control, there is increasing interest in combining systemic with regional corticosteroid techniques to mitigate chronic postsurgical pain. For example, the 'Prophylactic six-hourly intravenous acetaminophen to prevent postoperative delirium in older cardiac surgical patients' (PANDORA) trial reported promising outcomes using dexamethasone-palmitate-augmented parasternal nerve blocks to reduce long-term pain after sternotomy [12].

Mechanistically, dexamethasone’s analgesic effects are likely multifactorial, involving suppression of proinflammatory cytokines (e.g., interleukin-6, tumor necrosis factor (TNF)-α), attenuation of nociceptive signaling, inhibition of N-methyl-D-aspartate (NMDA) receptor activation, and modulation of glial cell activity in central pain pathways [5]. These mechanisms may help explain the downward trend in chronic pain at three months despite the modest dosing range used.

This study has several limitations that merit consideration. The retrospective, non-randomized design introduces potential selection bias and confounding, despite efforts to balance baseline characteristics between groups. The modest sample size (n = 45) limits statistical power, particularly for detecting differences in secondary outcomes such as chronic pain incidence or rare adverse events. Additionally, as a single-center study, the findings may not be generalizable to institutions with different anesthetic protocols, surgical techniques, or patient populations.

While the timing of dexamethasone administration was standardized, the use of a dosing range (0.1-0.2 mg/kg) introduces some heterogeneity that could influence the magnitude of the analgesic effect. The study also focused on subjective pain assessments using the Visual Analog Scale (VAS) at two postoperative time points, which, although validated, may not fully capture the complexity of pain evolution, long-term discomfort, or functional recovery.

Furthermore, the lack of serial inflammatory biomarker measurements (e.g., C-reactive protein, interleukin-6) and imaging data (e.g., CT scan evaluations) limits the ability to correlate analgesic outcomes with underlying physiological responses to inflammation or tissue healing. In addition, perioperative glycemic control was not systematically monitored, leaving potential metabolic side effects of corticosteroid use unexplored.

Despite these limitations, the findings support the hypothesis that early intraoperative dexamethasone administration enhances postoperative analgesia and reduces opioid consumption in cardiac surgery patients. The observed reductions in pain scores and morphine requirements suggest a clinically meaningful benefit.

To confirm these results and better define the role of dexamethasone in perioperative care, future prospective randomized controlled trials are warranted. Such studies should employ standardized corticosteroid dosing protocols, incorporate comprehensive multimodal pain assessments, include long-term follow-up, and evaluate inflammatory biomarkers, metabolic profiles, and imaging outcomes to fully assess both the efficacy and safety of perioperative steroid administration across diverse surgical populations.

## Conclusions

This retrospective cohort study provides meaningful insight into the role of intraoperative dexamethasone in optimizing postoperative pain management in cardiac surgery. Our findings demonstrate that a single, weight-adjusted IV dose administered after induction resulted in a significant reduction in opioid requirements and pain scores during the critical early recovery phase. The magnitude and consistency of these effects, reflected by a 34% reduction in morphine use and clinically relevant improvements in patient-reported pain, highlight the utility of dexamethasone as an effective adjunct within multimodal analgesia protocols. Although our study was not powered to detect statistical significance for long-term outcomes, the observed trend toward reduced chronic post-sternotomy pain at three months suggests a potentially durable benefit that warrants further exploration. Importantly, the intervention was delivered within a standardized perioperative framework without apparent safety concerns. These results support the integration of intraoperative dexamethasone as a pragmatic, low-cost strategy to enhance postoperative recovery, reduce opioid burden, and improve patient comfort following cardiac surgery.

## Appendices

Appendix A

Table 4 features raw data of the dexamethasone treatment group and controls.

**Table 4:** Raw data displaying the effects of dexamethasone on postoperative pain in cardiac surgery POD: Postoperative day, VAS: Visual analog scale

Patient ID	Group	Morphine on POD1 (mg)	Morphine on POD3 (mg)	VAS POD1	VAS POD3	Chronic pain three months postoperatively
P01	Dexamethasone	10	4	3	1	No
P02	Dexamethasone	12	5	4	2	No
P03	Dexamethasone	8	3	3	1	No
P04	Dexamethasone	11	4	4	1	No
P05	Dexamethasone	9	3	3	1	No
P06	Dexamethasone	10	4	3	2	No
P07	Dexamethasone	13	5	4	2	No
P08	Dexamethasone	12	5	4	2	No
P09	Dexamethasone	11	4	4	1	No
P10	Dexamethasone	10	4	3	2	No
P11	Dexamethasone	9	3	3	1	No
P12	Dexamethasone	8	3	2	1	No
P13	Dexamethasone	11	5	4	2	No
P14	Dexamethasone	10	4	3	2	No
P15	Dexamethasone	12	5	4	2	No
P16	Dexamethasone	9	3	2	1	No
P17	Dexamethasone	10	4	3	1	No
P18	Dexamethasone	11	4	3	2	No
P19	Dexamethasone	12	5	4	2	No
P20	Dexamethasone	13	6	5	3	No
P21	Dexamethasone	9	4	3	2	Yes
P22	Dexamethasone	8	3	2	1	Yes
P23	Control	14	6	5	3	No
P24	Control	15	7	6	3	No
P25	Control	13	6	5	2	No
P26	Control	16	8	6	4	No
P27	Control	14	7	5	3	No
P28	Control	15	6	6	3	No
P29	Control	13	7	5	3	No
P30	Control	14	7	5	4	No
P31	Control	16	8	6	4	No
P32	Control	17	8	6	3	No
P33	Control	15	7	5	3	No
P34	Control	14	7	6	3	No
P35	Control	16	8	6	4	No
P36	Control	13	7	5	3	No
P37	Control	14	6	5	3	No
P38	Control	15	7	6	3	Yes
P39	Control	17	9	7	4	Yes
P40	Control	18	8	6	4	Yes
P41	Control	16	8	6	4	Yes
P42	Control	15	7	5	3	Yes
P43	Control	17	9	6	4	Yes
P44	Control	16	7	6	4	Yes
P45	Control	15	6	5	3	Yes

## References

[1] Mueller XM, Tinguely F, Tevaearai HT, Revelly JP, Chioléro R, von Segesser LK (2000). Pain location, distribution, and intensity after cardiac surgery. Chest.

[2] Kleiman AM, Sanders DT, Nemergut EC, Huffmyer JL (2017). Chronic Poststernotomy pain: Incidence, risk factors, treatment, prevention, and the anesthesiologist’s role. Reg Anesth Pain Med.

[3] Barr LF, Boss MJ, Mazzeffi MA, Taylor BS, Salenger R (2020). Postoperative multimodal analgesia in cardiac surgery. Crit Care Clin.

[4] Chaney MA (2002). Corticosteroids and cardiopulmonary bypass : a review of clinical investigations. Chest.

[5] Murphy GS, Sherwani SS, Szokol JW (2011). Small-dose dexamethasone improves quality of recovery scores after elective cardiac surgery: a randomized, double-blind, placebo-controlled study. J Cardiothorac Vasc Anesth.

[6] Waldron NH, Jones CA, Gan TJ, Allen TK, Habib AS (2013). Impact of perioperative dexamethasone on postoperative analgesia and side-effects: systematic review and meta-analysis. Br J Anaesth.

[7] Dieleman JM, Nierich AP, Rosseel PM (2012). Intraoperative high-dose dexamethasone for cardiac surgery: a randomized controlled trial. JAMA.

[8] Myles PS, Dieleman JM, Munting KE (2024). Dexamethasone for cardiac surgery: a practice preference-randomized consent comparative effectiveness trial. Anesthesiology.

[9] Laconi G, Coppens S, Roofthooft E, Van De Velde M (2024). High dose glucocorticoids for treatment of postoperative pain: a systematic review of the literature and meta-analysis. J Clin Anesth.

[10] van Osch D, Dieleman JM, Nathoe HM (2015). Intraoperative high-dose dexamethasone in cardiac surgery and the risk of rethoracotomy. Ann Thorac Surg.

[11] Lee B, Schug SA, Joshi GP, Kehlet H (2018). Procedure-specific pain management (prospect) — an update. Best Pract Res Clin Anaesthesiol.

[12] Zhang H, Zhang T, Zheng Z (2025). Rationale and design for the thoracic Paravertebral Adjunctive Dexamethasone Palmitate Reducing chronic pain After cardiac surgery (PANDORA) trial: a parallel-group, double-blinded, randomised controlled, single-centre study. BMJ Open.

